# Budget impact analysis of the use of Souvenaid in patients with prodromal Alzheimer’s Disease in Spain

**DOI:** 10.1186/s13195-022-01111-7

**Published:** 2022-11-12

**Authors:** Javier Mar, Oliver Ibarrondo, Igor Larrañaga, Lorea Mar-Barrutia, Myriam Soto-Gordoa

**Affiliations:** 1 Basque Health Service (Osakidetza), Debagoiena Integrated Healthcare Organisation, Research Unit, Arrasate-Mondragón, Guipúzcoa, Spain; 2 Kronikgune Institute for Health Service Research, Barakaldo, Spain; 3 Biodonostia Health Research Institute, Donostia-San Sebastián, Guipúzcoa, Spain; 4 Unidad de Gestión Sanitaria, Hospital ‘Alto Deba’, Avenida Navarra 16, 20500 Mondragón, Spain; 5 Department of Psychiatry, Osakidetza Basque Health Service, Araba University Hospital, Vitoria-Gasteiz, Spain; 6 Faculty of Engineering, Electronics and Computing Department, Mondragon Unibertsitatea, Mondragon, Gipuzkoa, Spain

**Keywords:** Prodromal Alzheimer’s disease, Souvenaid, Budget impact analysis, Dementia, Clinical Dementia Rating

## Abstract

**Introduction:**

The effectiveness, safety, and cost-effectiveness of the use of Souvenaid for Alzheimer’s disease (AD) have been previously evidenced. To complete the economic analysis, there is a need to assess whether society can afford it. The objective of this study was to carry out a budget impact analysis of the use of Souvenaid in Spain under the conditions of the LipiDidiet clinical trial from a societal perspective.

**Methods:**

We built a population model that took into account all the cohorts of individuals with AD, their individual progression, and the potential impact of Souvenaid treatment on their trajectories. Patient progression data were obtained from mixed models. The target population was estimated based on the population forecast for 2020–2035 and the incidence of dementia. Individual progression to dementia measured by the Clinical Dementia Rating-Sum of Boxes was reproduced using mixed models. Besides the costs of treatment and diagnosis, direct costs of medical and non-medical care and indirect costs were included.

**Results:**

The epidemiological indicators and the distribution of life expectancy by stages validated the model. From the third year (2022), the differences in the cost of dementia offset the incremental cost of diagnosis and treatment. The costs of dependency reached €500 million/year while those of the intervention were limited to €40 million.

**Conclusions:**

Souvenaid, with modest effectiveness in delaying dementia associated with AD, achieved a positive economic balance between costs and savings. Its use in the treatment of prodromal AD would imply an initial cost that would be ongoing, but this would be offset by savings in the care system for dependency associated with dementia from the third year. These results were based on adopting a societal perspective taking into account the effect of treatment on the use of health, social, and family resources.

**Supplementary Information:**

The online version contains supplementary material available at 10.1186/s13195-022-01111-7.

## Introduction

Dementia remains one of the leading causes of morbidity and mortality worldwide [1], and the future is daunting as the “baby boom” generation is reaching older ages [2]. Already, around 40–50 million people globally are living with dementia, and the figure is forecasted to double every two decades, being expected to exceed 100 million by 2050 [3, 4]. The prevalence of Alzheimer’s disease (AD), the most common type of dementia, contributes to 50–75% of all dementia cases [5]. Dementia has a profound negative impact on families, communities, and healthcare systems alike [6]. Arising from both the direct medical and social care costs as well as the cost of informal care, the financial implications of the condition are equivalent to 1.1% of the global gross domestic product [7].

Currently, there is no AD disease-modifying therapy on the market, but 80% of AD drugs in development are disease-modifying non-drug and 20 drug candidates are in phase III development [8]. There are also a number of non-drug interventions that have demonstrated statistically and clinically meaningful benefits for people living with AD and their caregivers, including improving quality of life [9, 10]. Souvenaid is a medical food that targets early symptoms such as the loss of episodic memory during the mild cognitive impairment (MCI) stage, which is associated with synaptic abnormalities [11, 12]. It contains a combination of nutrients called Fortasyn Connect, which includes precursors and cofactors necessary for forming neuronal membranes that hypothetically serve to support the synthesis of new synapses and maintain existing ones [13]. The LipiDiDiet clinical trial demonstrated better outcomes in neuropsychological tests in patients who received Souvenaid [14] with a 3-year follow-up. Some improvements, such as in the Clinical Dementia Rating Sum of Boxes (CDR-SB) score and the atrophy rate on magnetic resonance imaging (MRI), were already detected after the second year [15]. These data are encouraging since delaying dementia is a relevant result as it would significantly reduce the population burden of AD [16]. The previous analysis performed showed that despite the only modest improvement in the course of the disease, as dementia costs are high, the intervention was cost-effective compared to placebo [17]. In the base case scenario, in which Souvenaid was administered during the MCI stage and the costs of diagnosis were included, the treatment was considered cost-effective from a societal perspective. It had an incremental cost-utility ratio (ICUR) of €22,743/quality-adjusted life year (QALY), well below the willingness-to-pay thresholds in Spain (€25,000/QALY) [18] and within the range of the National Institute for Health and Care Excellence (NICE) (£20,000 to £30,000/QALY) [19].

The reality is that healthcare payers impose financial constraints on the use of new treatments [20]. Therefore, in addition to cost-effectiveness analysis, it is necessary to consider the budgetary impact on decision-making [21]. Most value frameworks focus specifically on the initial adoption/funding decisions and have limited mechanisms for evaluating value over time [22]. This matters in the case of AD, as it is a neurodegenerative disease, and people with AD can live many years after becoming dependent. Interventions may have long-term effects, but clinical trials of AD interventions tend to have only relatively short follow-up periods. Mathematical models can help estimate long-term impacts [23]. This dimension of the decision-making process is covered by the inclusion of budget impact analysis (BIA). As explained by Mauskopf et al., “BIA is an essential part of a comprehensive economic assessment of a health-care technology and is increasingly required, along with cost-effectiveness analysis (CEA), before formulary approval or reimbursement” [24].

The objective of this study was to carry out a BIA of Souvenaid in Spain under the conditions of the LipiDidiet clinical trial for the years 2020–2035. BIAs usually take the perspective of the payer, typically the health system [21]. Yet, adopting a healthcare system perspective ignores societal costs such as the impact on caregivers, which is one of the largest cost drivers of AD [22]. Therefore, this study was performed from a social perspective.

## Methods

A BIA [25] was performed in Excel using Visual Basic for Applications (VBA) coding to estimate the potential economic impact of using Souvenaid in the Spanish population. The analysis consists of comparing a scenario with Souvenaid against placebo. Three main components are taken into account: the target population, the market penetration of Souvenaid, and the costs and savings associated with the treatment. For the purpose of this study, Souvenaid’s market share was established at 7.5%.

### Target population

We considered as the target population only 7.5% of the total Spanish population with prodromal AD from 2020 to 2035. Prodromal AD incidence rates were estimated from the Spanish real-world data dementia registries (Additional file 1: Table S1), which have shown great consistency with the Basque and Catalan populations with prodromal AD [26, 27]. Since the registries did not differentiate between the types of dementia, it was assumed that AD represented two-thirds of the total incidence of dementia. Based on the dementia incidence rates by age and sex [26] and the expected Spanish population for the years 2020–2035 according to the National Institute of Statistics (INE) [28], the target population for each year was estimated by adjusting the starting age for the duration of the MCI stage [17].

### The conceptual model for the BIA

The simulation model built represented the natural history of AD starting from the prodromal stage. For that purpose, we introduced all the cohorts of individuals with prodromal AD from 2020 to 2035 assigned to receive Souvenaid. We made our analysis assuming a 7.5% market penetration. Each entity in the model was assigned individual characteristics such as age, sex, and baseline Mini-Mental State Exam (MMSE) and CDR-SB scores. This population was cloned to explore the differences in disease progression based on whether people received Souvenaid or placebo. According to individual characteristics and treatment strategy, patients were assigned moments at which they progressed to the different AD stages, namely, mild or moderate AD and death. This information enabled us to determine the number of individuals in each of the stages from 2020 onwards. Based on the 7.5% market penetration of patients receiving the treatment of interest in the prodromal stage, the prevalence of mild and moderate AD, and the costs associated with this treatment and the disease itself, the budget impact was calculated. Treatment discontinuity was out of scope due to a lack of information.

### Time horizon

As with other neurodegenerative diseases, people with AD may live many years beyond becoming dependent. This survival may be as long as 7 to 10 years in those with an onset of AD at the age of 70. Therefore, it is crucial to take a long-term perspective when evaluating interventions targeting AD [22, 29]. As the introduction of new cohorts can blur the long-term effects of an intervention, we included new cohorts from 2020 to 2035 but followed the individuals that were still alive until 2040.

### Parameters

Parameters used to populate the model are listed in Table 1 (basic parameters) and Additional file 1: Table S2 (basic and statistical parameters). As mentioned in the target population section, age was based on the Spanish population’s prodromal AD incidence rates. Clinical characteristics of the population were defined according to the features of the LipiDiDiet control group [15]. The attributes considered were sex and baseline MMSE and CDR-SB scores, assuming these scores to be correlated based on data in the literature [30].

**Table 1:** Budget impact analysis model parameters

		Source
**Model characteristics**		
Number of patients	89,030	
Number of replications	1	
Cutoff for mild AD	4.5	[31]
Cutoff for moderate AD	9.5	[31]
**Population characteristics**		
Population projections by age group (2020–2035)	INE	
Incidence rates	Literature	[26]
Age of the caregiver (mean)	55.2	[15]
Baseline CDR-SB score (mean)	1.76	[15]
Baseline MMSE score (mean)	26.9	[15]
**Annual costs** (m**ean**)		[32] payer
Treatment with Souvenaid (per year)	€1200	Patient
Diagnosis	€284	Health service
Direct healthcare for mild AD	€3388	Health service
Direct social care for mild AD	€13,927	F&SS
Indirect for mild AD	€647	
Total for mild AD	€17,962	
Direct healthcare for moderate/severe AD	€4659	Health service
Direct social care for moderate/severe AD	€34,893	F&SS
Indirect for moderate/severe AD	€820	
Total for moderate/severe AD	€40,372	

*AD* Alzheimer’s disease, *CDR-SB* Clinical Dementia Rating-Sum of Boxes, *MMSE* Mini-Mental State Exam, *F&SS* Family &Social Services

Progression to dementia as measured by the CDR-SB was estimated using the mixed model constructed by Van Oudenhoven et al. (see Additional file 1: Table S2) from the results of the LipidDidiet trial at 2 years for both the control and intervention groups [15, 33]. The cutoff points used for dementia were 4.5 for mild and 9.5 for moderate to severe disease [34]. Based on the LipidDidiet trial, the CDR-SB progressed from baseline to month 24 a mean change of 1.12 (SD1.72) in the control group and 0.56 (SD1.32) in the intervention group. Taking into account that difference in progression, the competitive risk of death, and the incorporation of new cohorts, the model supplied the new epidemiological scenario and calculated the difference in incidence and prevalence of dementia. Multiplied by the unit costs, these epidemiological indicators were the base for the BIA.

Time until death by other causes was assigned using a specific Gompertz function for each sex [17]. Patients were assigned no excess risk of death during the prodromal stage but were assigned a specific risk of death when they reached the mild dementia stage [35].

### Costs

As in any disease that generates disability, various public services face different but complementary tasks in providing care. In particular, while health services assume the short-term cost of new AD treatments, delaying dementia means savings in social services as well as in informal costs borne by families [36]. As noted above, it is for this reason that we took a societal perspective and considered both direct and indirect costs (Table 1). In particular, we incorporated the following costs: the costs of the intervention including both the diagnosis and the treatment, and the costs of mild and moderate stages of dementia as reported in the literature.

The cost of diagnosis per patient in the literature is €2900 for a diagnostic process that includes a primary care consultation, a neurology consultation, a neuropsychological examination, an MRI scan, and a cerebrospinal fluid (CSF) biomarker test [37]. On the other hand, we understood that all the components except testing for CSF biomarkers may be part of the processes of care for people with MCI, and therefore, the increase in diagnostic cost should be limited to the last item. The cost for CSF biomarkers was estimated at €284, the sum of the cost of lumbar puncture (€158) [38], and that of measuring the levels of four relevant biomarkers (namely, tau, phosphorylated tau, and amyloid beta peptides 40 and 42), which was €126 in the reference laboratory of the Basque Country. Regarding the treatment, Souvenaid was administered only during the MCI stage and at a cost of €1200 per year. Currently, Souvenaid is not reimbursed.

The costs of mild and moderate AD stages were obtained from López-Bastida et al. [32]. In that study, the authors used a prevalence-based approach to estimate the cost of AD in Spain. They accounted for the direct costs derived from medical and non-medical care and the indirect costs obtained through the use of the human capital theory [32, 39, 40] that are associated with the presence of dementia for 1 year. Unit costs were updated to 2019 to take into account inflation from the year in which they were calculated, but as recommended in ISPOR guidelines for BIA, no discount was applied [21]. Table 1 also illustrates the cost by payer. The Health Service covers the diagnosis and medical costs of dementia, family and Social Services cover direct social costs, and the patient’s the ones for treatment.

### Model validation

We used the following parameters to assess the model’s accuracy in reproducing the natural history of AD: patients’ characteristics such as age, baseline CDR-SB, and MMSE scores and their life expectancy [15, 35] and the yearly (2020–2035) incidence and prevalence of the disease [26].

## Results

### Validation results

Baseline MMSE and CDR-SB scores were 26.94 (SD = 5.24) and 1.76 (SD = 1.14), respectively, which are similar to the values reported by Soininen et al. [15]. On the other hand, the simulated population was older than the LipiDiDiet clinical trial population (78.97 [SD = 8.53] versus 70.7 [SD = 6.2] years). The overall life expectancy was estimated at 8.87 (SD = 5.54) years distributed into 3.76 years with MCI and 5.11 years with dementia. The latter is consistent with the results obtained by Dodge [35] who estimated a 5.2-year survival for 78-year-old individuals with dementia. Individuals in the intervention group were estimated to have a slightly longer overall survival (9.98 [SD = 6.42]; corresponding to 4.90 [SD = 5.45] years with MCI and 5.08 [SD = 3.12] years with dementia).

Table 2 lists the epidemiological results of the model. These are the incidence and prevalence of dementia in control and intervention groups. By the time the model reached a steady state in 2030, the incidence of dementia had risen to 6700 cases and the prevalence of dementia to 22,200 cases. Taking into account the annual inclusion of new cohorts until 2035 and the trajectories of the individuals who progress to dementia (CDR-SB > 4.5) in the two groups (control and intervention), the model calculated the dementia incidence and prevalence figures shown in this table.

**Table 2:** Epidemiological results of the model from 2020 to 2040 with both alternatives

Year	Control		Intervention	
	Dementia incidence	Dementia prevalence	Dementia incidence	Dementia prevalence
2020	948	435	672	291
2021	2017	1779	1505	1239
2022	2939	3850	2234	2766
2023	3853	6572	3099	4818
2024	4623	9503	3692	7177
2025	5328	12,503	4217	9608
2026	5721	15,209	4709	11,933
2027	6043	17,532	4953	13,946
2028	6297	19,420	5239	15,661
2029	6539	20,865	5417	17,053
2030	6786	22,220	5779	18,280
2031	7032	23,424	5851	19,518
2032	6999	24,427	6087	20,457
2033	7370	25,320	6228	21,339
2034	7401	26,075	6525	22,186
2035	7956	26,958	6665	23,075
2036	6686	27,289	6093	23504
2037	5337	26,168	5104	22,953
2038	4279	23,961	4064	21,465
2039	3183	21,013	3278	19,341
2040	2161	17,659	2485	16,801

### Budget impact analysis results

The budget impact analysis results are presented in Table 3.

**Table 3:** Total budget impact analysis in millions of euros from 2020 to 2040 of Souvenaid from 2020 to 2035 being the health service the payer for diagnosis and dementia medical costs, the Family and Social Services for direct social costs, and the patient for treatment

Year	Control group			Intervention group				
	Mild dementia	Moderate dementia	Total	Treatment	Diagnosis	Mild dementia	Moderate dementia	Total
2020	7.65	0.23	**7.88**	5.40	1.36	5.11	0.17	**12.05**
2021	30.95	1.48	**32.43**	10.04	1.39	21.17	1.58	**34.19**
2022	66.25	4.27	**70.51**	13.94	1.41	46.50	4.68	**66.53**
2023	111.35	9.83	**121.18**	17.08	1.44	79.57	10.22	**108.31**
2024	157.13	19.91	**177.04**	19.66	1.46	115.86	19.16	**156.14**
2025	199.76	36.42	**236.19**	21.89	1.49	151.67	30.70	**205.75**
2026	235.88	54.74	**290.62**	23.83	1.52	183.44	45.34	**254.13**
2027	265.30	72.82	**338.12**	25.70	1.55	209.78	59.78	**296.80**
2028	289.59	86.95	**376.54**	27.46	1.58	231.81	72.64	**333.48**
2029	306.48	100.24	**406.72**	29.24	1.61	248.92	84.24	**364.01**
2030	323.55	110.91	**434.46**	30.93	1.65	264.50	93.72	**390.80**
2031	339.18	119.72	**458.90**	32.52	1.69	280.65	102.65	**417.51**
2032	352.70	126.32	**479.01**	34.24	1.72	292.41	110.15	**438.52**
2033	363.47	134.05	**497.53**	35.99	1.76	302.46	118.65	**458.86**
2034	372.31	140.99	**513.30**	37.66	1.80	313.45	124.84	**477.75**
2035	384.33	146.61	**530.94**	39.36	1.84	324.58	131.95	**497.73**
2036	386.61	152.01	**538.61**	33.53	-	327.49	138.99	**500.00**
2037	364.71	154.60	**519.31**	28.64	-	314.10	144.11	**486.86**
2038	326.06	153.13	**479.19**	24.98	-	286.85	144.89	**456.72**
2039	276.20	148.60	**424.80**	22.22	-	250.40	142.38	**415.00**
2040	223.63	137.33	**360.96**	20.18	-	209.72	135.13	**365.03**

**Fig. 1:**
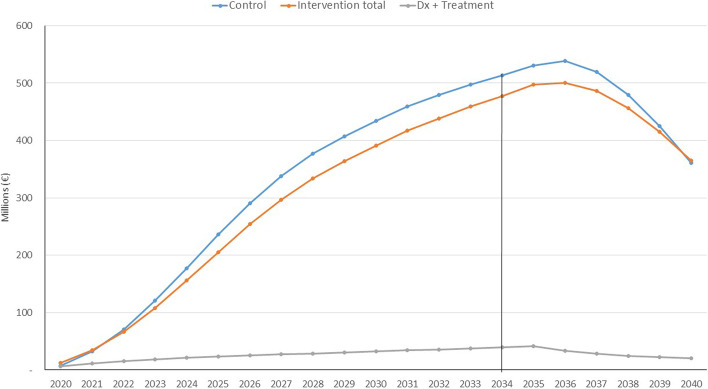
Budget impact analysis from 2020 to 2040 associated with the use of Souvenaid from 2020 to 2035

Table 3, Additional file 1: Table S3, and Fig. 1 show the BIA results broken down by cost of diagnosis, treatment, and stage of disease (mild or moderate dementia) for the control and intervention groups, being the Health Service the payer for diagnosis and dementia medical costs, the Family and Social Services for direct social costs and the patient for treatment. The cost of diagnosis in the intervention accounted for the cost overrun with respect to the control group. The control group received no treatment, and therefore, this cost was omitted. Although in the first few years, costs were slightly higher in the intervention group than in the control group, this trend changed from the third year (2023) onwards. The explanation lies in the level of the diagnosis and treatment costs, these representing less than 10% of the total costs in the intervention group, as can be seen in Fig. 1. This figure vividly illustrates the large difference in magnitude between the costs of dependency, which reach €500 million, and those of the intervention, which reach a maximum of €40 million.

**Fig. 2:**
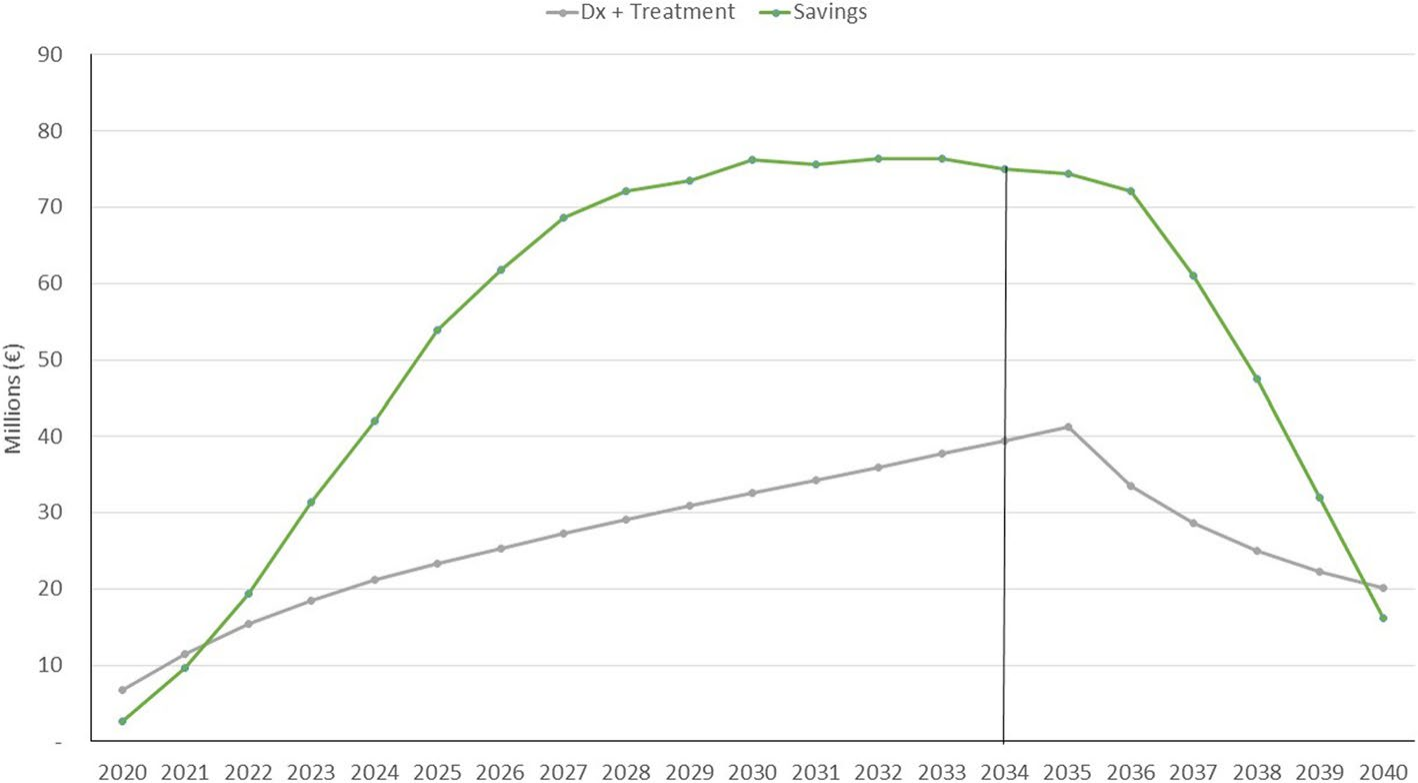
Incremental costs and savings from 2020 to 2040 associated with the use of Souvenaid from 2020 to 2035

The relationship between the incremental costs due to diagnosis and treatment and the savings due to the lower prevalence of dementia is shown in Fig. 2. This figure helps us to visualize the process of incorporating new cohorts into the population model and how there are net savings from 2022 due to the reduction in dementia. It also makes it possible to compare the different roles of the health system as a possible funder of diagnosis and treatment and of social services and families that bear the burden of dementia.

## Discussion

This study has shown that the use of a nutritional supplement such as Souvenaid that achieves a delay in the onset of AD-related dementia could produce up to €40 million annual savings in the Spanish population if the intervention were to be well established for 7.5% of the AD population. As the BIA is proportional to the incidence and the percentage of market uptake, extrapolating to the 100% of the incident AD patients would render €533 million of savings. Currently, this percentage is very small, and its increase or reduction will depend on the evidence collected in trials such as LipiDidiet. Nonetheless, society would not be able to see any savings until the third year (2023), when the delay in dementia would start to have an impact on its prevalence. Despite the modest effects found in the clinical trial, the relatively low cost of the treatment and the high social and family costs of dependency means that this treatment could have a major economic impact.

Analyzing the affordability of Souvenaid treatment complements the results of studies reporting its effectiveness in the LipiDiDiet clinical trial, specifically, significant benefits in cognition, measures of brain volume, and the performance of daily living tasks as measured by the CDR-SB [14, 15]. Our model of trajectories was based on this last measure, namely, score on the CDR-SB, which has been described as a sensitive and significant clinical outcome for clinical trials with patients in prodromal AD [41]. Models applied for the economic evaluation of aducanumab also used it to reproduce patients’ trajectories [42]. The budgetary impact of Souvenaid is sensitive to the inclusion of the cost of the diagnosis if this is taken to include all the components of the first contact of patients with the health system leading to the diagnosis [37]. Incorporating biomarkers into neurological practice is key to responding to the challenge of defining the target population that will benefit from any given intervention [37]. The lack of a disease-modifying drug does not justify a fatalistic approach to clinical care for people with MCI. On the contrary, a reduction in the population burden of AD may well be achieved not so much by the appearance of a disease-modifying and disruptive treatment but by the summing of multiple actions with small or moderate effects. The recommendation to anticipate the diagnosis of AD at the prodromal stage using biomarkers seeks to make it easier for patients to organize their personal future and enable the use of interventions that delay its progression. Treatment with Souvenaid can be combined with intensive programs to reduce risk factors for AD such as FINGER which have shown modest but significant benefits [43].

The idea that a treatment may be cost-effective, but cannot be adopted due to its budgetary impact, was raised a few years ago concerning hepatitis C [44] and has appeared more recently in the field of AD [45]. In 2015, the evaluation of hepatitis C treatments was summarized paradoxically as “cost-effective but unaffordable” [44, 46, 47]. Over the subsequent years, the reality has been different, most of the millions of patients with hepatitis C in Europe having eventually been treated, and the debate is now focused on its eradication [48]. The impossible became possible because the price per patient for the treatments fell from an initial €100,000 [47] to €18,000 [46] and because their added health benefits, specifically, avoiding the progression towards chronic liver disease, were taken into account [49].

We wonder whether a similar process may occur in the treatment of AD. Given its prevalence, AD treatment will be impossible to afford if the cost per patient is $56,000 per year [50]. When it is said that a health system cannot pay for a treatment option despite it being cost-effective, what is meant is that the “threshold” used to judge profitability does not properly reflect the value of the benefits of the treatment for the disease of interest [44]. In the case of AD, the undervalued component is the dependency associated with dementia. Whittington et al. estimated that the price of aducanumab would need to drop to a tenth of the annual price of $56,000 to become cost-effective for the American threshold of $100,000 per QALY gained [42]. If they had considered European willingness to pay thresholds, which lie in a range from €25,000 to €50,000, an even greater reduction would have been needed [18].

In Europe, healthcare and social care costs are usually represented as unconnected silos, which makes it difficult to assess the real economic impact of AD. The same barrier exists in the USA between the financing of healthcare by Medicare or private insurers and that of long-term care that falls largely on families themselves (informal care and out-of-pocket costs) [45, 51]. Specifically, it is necessary to stop viewing informal care provided by families as a low-cost service, without taking into account the consequences for caregivers in health and quality of life [52].

This study does not address the economic impact of population screening for MCI since it is not recommended due to the lack of straightforward diagnostic methods and the consequence of many false positives or overdiagnoses [53]. The clinical course of the target population analyzed in this study started when the patients made contact with the health system due to memory loss and having been evaluated by neuropsychological tests joined the category of individuals diagnosed with MCI. In addition, to make the diagnosis of prodromal AD, it is required that a lumbar puncture is carried out by a neurologist and CSF biomarkers are found to be positive [37]. The epidemiological model was based on the arbitrary assumption that the number of patients treated corresponded to 7.5% of the cases of incident dementia due to AD in Spain. As the costs of the intervention and the savings from dementia averted are proportional to the size of the population, the results can be projected to any percentage of the total population with prodromal AD. In reality, what is most relevant is not so much the magnitude of these two figures (costs and savings) but the relationship between them.

Wimo et al. made several recommendations for the economic evaluation of AD that we have followed [37]. First, the progression of the disease was integrated into an epidemiological model with population data converting the surrogate effect of the intervention measured in the clinical trial into progression to dementia [14, 15, 17]. Second, the costs included the entire natural history of AD from the prodromal stage to the social costs of dementia [32]. A key component is the cost of the treatment, which is just €1200 per year.

### Limitations

It is necessary to point out the difficulty of matching the study population of the clinical trial with the profile of MCI patients demanding care from the health system. In our case, the study population was younger than the clinical practice patients. Since the mixed models function used in the model did not link CDR-SB progression to age, our results could be slightly biased [15, 26, 33]. Moreover, we have assumed the same delaying seeking medical attention in clinical practice to that in the trial notwithstanding that volunteers recruited for trials are typically more engaged in their health than members of the general population.

We conducted the BIA for the Spanish population. However, the mathematical model presented is based on the LipiDiDiet study, which recruited patients in Finland, Germany, the Netherlands, and Sweden. We acknowledge that assuming the intervention will have the same benefits in the Spanish population posits a limitation.

Incorporating a partial effect on patient progression due to treatment withdrawal was out of range due to a lack of information. This should be addressed whenever such evidence become available.

## Conclusions

We conclude that Souvenaid, with modest effectiveness in delaying dementia associated with AD, achieved a positive economic balance between costs and savings in the medium term. Its use in the treatment of prodromal AD would imply an initial cost that would be ongoing, but this would be offset by savings in the care system for dependency associated with dementia from the third year. These results were based on adopting a societal perspective that considers the effect of treatment on the use of health, social, and family resources.

## Supplementary Information

**Additional file 1: Table S1.** Dementia incidence with confidence intervals (CIs). Obtained from reference 25. Mar J, Gorostiza A, Arrospide A, Larrañaga I, Alberdi A, Cernuda C, et al. Estimation of the epidemiology of dementia and associated neuropsychiatric symptoms by applying machine learning to real-world data. Rev Psiquiatr Salud Ment. 2021; S1888-9891(21)00032-X. **Table S2.** Model parameters. **Table S3.** Total budget impact analysis in millions of euros with confidence intervals from 2020 until 2040 associated with the use of Souvenaid from 2020 to 2035. CI: confidence interval.

## Data Availability

No dataset has been used, and all the parameters are shown in the article.
