# Supplementary material for: Budget impact analysis of the use of Souvenaid in patients with prodromal Alzheimer’s Disease in Spain
Source: Alzheimers Res Ther. 2022 Nov 12;14:171. doi: 10.1186/s13195-022-01111-7 (PMC9652901; doi:10.1186/s13195-022-01111-7)
Supplement: Supplementary file 1 — Additional file 1: Table S1. Dementia incidence with confidence intervals (CIs). Obtained from reference 25. Mar J, Gorostiza A, Arrospide A, Larrañaga I, Alberdi A, Cernuda C, et al. Estimation of the epidemiology of dementia and associated neuropsychiatric symptoms by applying machine learning to real-world data. Rev Psiquiatr Salud Ment. 2021; S1888-9891(21)00032-X. Table S2. Model parameters. Table S3. Total budget impact analysis in millions of euros with confidence intervals from 2020 until 2040 associated with the use of Souvenaid from 2020 to 2035. CI: confidence interval. [file 13195_2022_1111_MOESM1_ESM.docx]

**Supplementary material Additional file 1**

**Table SM1**. Dementia incidence with confidence intervals (CIs).

| Age group | Incidence x 1000 | Lower CI | Upper CI |
| --- | --- | --- | --- |
| Total | 6.8 | 6.6 | 7.0 |
| [60;64) | 0.4 | 0.3 | 0.5 |
| [65;69) | 1.0 | 0·8 | 1.2 |
| [70;74) | 3.6 | 3.3 | 4.0 |
| [75;79) | 9.5 | 8.8 | 10.2 |
| [80;84) | 16.3 | 15.3 | 17.3 |
| [85;89) | 23.9 | 22.5 | 25.3 |
| [90;105) | 25.2 | 23.2 | 27.3 |

Obtained from reference 25. Mar J, Gorostiza A, Arrospide A, Larrañaga I, Alberdi A, Cernuda C, et al. Estimation of the epidemiology of dementia and associated neuropsychiatric symptoms by applying machine learning to real-world data. Rev Psiquiatr Salud Ment. 2021; S1888-9891(21)00032-X.

**Table SM2**. Model parameters

| **Time to death due to other causes** | | | |  |
| --- | --- | --- | --- | --- |
|  | Male | Alfa | 6.9E-05 | Gompertz |
|  |  | Beta | 0.087 |  |
|  | Female | Alfa | 3.8E-05 |  |
|  |  | Beta | 0.084 |  |
| **CDR-SB evolution (mixed models)** | | |  | [31] |
|  | β_0_ | Mean (SD) | 4.6586 (0.4107) |  |
|  | β_1_ | Mean (SD) | 0.609 (0.047) |  |
|  | β_2_ | Mean (SD) | 0.071 (0.077) |  |
|  | β_3_ | Mean (SD) | -0.233 (0.069) |  |
|  | β_4_ | Mean (SD) | -0.122 (0.015) |  |
|  | β_51_ | Mean (SD) | 0.6846 (0.0878) |  |
|  | β_52_ | Mean (SD) | 0.6069 (0.0947) |  |
|  | β_53_ | Mean (SD) | -0.4693 (0.0958) |  |
|  | β_54_ | Mean (SD) | 1.7528 (0.1037) |  |
|  | β5_5_ | Mean (SD) | 0.266 (0.1025) |  |
| **Survival with Alzheimer's disease** | | |  | [33] |
|  | Male |  |  |  |
|  | Intercept | Mean (SD) | -87.380 (32.716) |  |
|  | Age | Mean (SD) | 3.742 (1.156) |  |
|  | Age^2^ | Mean (SD) | -0.048 (0.014) |  |
|  | Age^3^ | Mean (SD) | 0.00 (0.00) |  |
|  | Female |  |  |  |
|  | Intercept | Mean (SD) | 111.800 (37.957) |  |
|  | Age | Mean (SD) | -2.560 (1.342) |  |
|  | Age^2^ | Mean (SD) | 0.019 (0.016) |  |
|  | Age^3^ | Mean (SD) | -4.25E-05 (6.08E-05) |  |

**Table SM3**. Total budget impact analysis in millions of euros with confidence intervals from 2020 until 2040 associated with the use of Souvenaid from 2020 to 2035.

|  |  | Control |  |  | Intervention |  |
| --- | --- | --- | --- | --- | --- | --- |
| Year | Mean | Lower CI | Upper CI | Mean | Lower CI | Upper CI |
| 2020 | 7,88 | 7,34 | 8,43 | 12,05 | 11,22 | 12,88 |
| 2021 | 32,43 | 30,19 | 34,67 | 34,19 | 31,83 | 36,54 |
| 2022 | 70,51 | 65,65 | 75,38 | 66,53 | 61,94 | 71,12 |
| 2023 | 121,18 | 112,82 | 129,54 | 108,31 | 100,84 | 115,79 |
| 2024 | 177,04 | 164,82 | 189,25 | 156,14 | 145,36 | 166,91 |
| 2025 | 236,19 | 219,89 | 252,48 | 205,75 | 191,55 | 219,94 |
| 2026 | 290,62 | 270,57 | 310,68 | 254,13 | 236,60 | 271,67 |
| 2027 | 338,12 | 314,79 | 361,45 | 296,80 | 276,32 | 317,28 |
| 2028 | 376,54 | 350,56 | 402,52 | 333,48 | 310,47 | 356,49 |
| 2029 | 406,72 | 378,66 | 434,78 | 364,01 | 338,90 | 389,13 |
| 2030 | 434,46 | 404,49 | 464,44 | 390,80 | 363,84 | 417,77 |
| 2031 | 458,90 | 427,24 | 490,57 | 417,51 | 388,70 | 446,32 |
| 2032 | 479,01 | 445,96 | 512,07 | 438,52 | 408,26 | 468,78 |
| 2033 | 497,53 | 463,20 | 531,85 | 458,86 | 427,20 | 490,52 |
| 2034 | 513,30 | 477,88 | 548,72 | 477,75 | 444,79 | 510,72 |
| 2035 | 530,94 | 494,30 | 567,57 | 497,73 | 463,39 | 532,08 |
| 2036 | 538,61 | 501,45 | 575,78 | 500,00 | 465,50 | 534,50 |
| 2037 | 519,31 | 483,47 | 555,14 | 486,86 | 453,27 | 520,45 |
| 2038 | 479,19 | 446,13 | 512,26 | 456,72 | 425,20 | 488,23 |
| 2039 | 424,80 | 395,49 | 454,11 | 415,00 | 386,37 | 443,64 |
| 2040 | 360,96 | 336,06 | 385,87 | 365,03 | 339,85 | 390,22 |

CI: confidence interval.
